# TOR signaling pathway and autophagy are involved in the regulation of circadian rhythms in behavior and plasticity of L2 interneurons in the brain of *Drosophila melanogaster*

**DOI:** 10.1371/journal.pone.0171848

**Published:** 2017-02-14

**Authors:** Ewelina Kijak, Elżbieta Pyza

**Affiliations:** Department of Cell Biology and Imaging, Institute of Zoology, Jagiellonian University, Kraków, Poland; Karlsruher Institut fur Technologie, GERMANY

## Abstract

*Drosophila melanogaster* is a common model used to study circadian rhythms in behavior and circadian clocks. However, numerous circadian rhythms have also been detected in non-clock neurons, especially in the first optic neuropil (lamina) of the fly’s visual system. Such rhythms have been observed in the number of synapses and in the structure of interneurons, which exhibit changes in size and shape in a circadian manner. Although the patterns of these changes are known, the mechanism remains unclear. In the present study, we investigated the role of the TOR signaling pathway and autophagy in regulating circadian rhythms based on the behavior and structural plasticity of the lamina L2 monopolar cell dendritic trees. In addition, we examined the cyclic expression of the TOR signaling pathway (*Tor*, *Pi3K class 1*, *Akt1*) and autophagy (*Atg5* and *Atg7*) genes in the fly’s brain. We observed that *Tor*, *Atg5* and *Atg7* exhibit rhythmic expressions in the brain of wild-type flies in day/night conditions (LD 12:12) that are abolished in *per*^*01*^ clock mutants. The silencing of *Tor* in *per* expressing cells shortens a period of the locomotor activity rhythm of flies. In addition, silencing of the *Tor* and *Atg5* genes in L2 cells disrupts the circadian plasticity of the L2 cell dendritic trees measured in the distal lamina. In turn, silencing of the *Atg7* gene in L2 cells changes the pattern of this rhythm. Our results indicate that the TOR signaling pathway and autophagy are involved in the regulation of circadian rhythms in the behavior and plasticity of neurons in the brain of adult flies.

## Introduction

Circadian rhythms observed in animal behavior and in the brain are generated by a circadian system composed of central (pacemaker) and peripheral clocks. The pacemaker of *D*. *melanogaster* consists of approximately 75 clock neurons in each brain hemisphere that cyclically express so-called “clock genes”. In both flies and mammals, the circadian rhythm in clock cells is generated by the molecular clock, which comprises transcriptional feedback loops [1, 2]. The key players in this mechanism of *D*. *melanogaster* are *period* (*per*) and *timeless* (*tim*) genes, their proteins PER and TIM, and the transcription factors CLOCK (CLK) and CYCLE (CYC). Their transcription is tightly controlled, but their posttranscriptional and posttranslational modifications are important for the clock and the generation of oscillations. At the end of day and at the beginning of the night CLK and CYC, as heterodimers, activate transcription of *per* and *tim*, and at the end of the night, their proteins accumulate and form PER-TIM heterodimers. Next, they enter the nucleus, bind to CLK-CYC heterodimers and inhibit transcription of their own genes. The next cycle starts when PER and TIM are degraded at the beginning of the day. The transcription factors CLK and CYC control the expression of not only clock genes but also clock-controlled genes, which are not part of the molecular clock but which have cyclic expression, and their proteins are involved in rhythmic processes in clock neurons and in other cells. Circadian information from the pacemaker is transmitted to target cells and tissues, which exhibit circadian rhythms in biochemical and physiological processes, and finally the rhythms are observed in the behavior of animals.

The most studied circadian rhythms are behavioral rhythms, especially in locomotor activity, however, the mechanisms of transmission of circadian information from the pacemaker to motor centers that regulate locomotor activity are mostly unknown. Another output system from the pacemaker has been detected in the visual system of *D*. *melanogaster*, particularly in the first optic neuropil (lamina). In the lamina, circadian plasticity has been observed in the size of neurons and glial cells and in the number of synapses (reviewed by [3, 4]). Moreover, circadian changes have been found in the level of the α subunit of Na^+^/K^+^-ATPase [5, 6] and in the abundance of the presynaptic scaffolding protein Bruchpilot (BRP) [7]. All rhythms in the lamina, including circadian neuroplasticity and the rhythms of the expression of specific genes and proteins are controlled by the central clock via the release of at least two clock neurotransmitters, pigment-dispersing factor (PDF) and ion transport peptide (ITP), and by peripheral clocks located in the retina and glial cells [6, 7, 8]. The rhythm that is particularly pronounced in the lamina of several fly species has been observed in the structure of first-order interneurons, L2 monopolar cells [9, 10, 11]. L2 monopolar cells are one of the five (L1-L5) types of monopolar cells that form regular cylindrical modules, called cartridges, with photoreceptor terminals and other cell types. They receive photic information from the retina photoreceptors R1-R6 by tetrad synapses, and L2 is one of the four postsynaptic cells in those synaptic contacts. Their somata are located in the distal lamina, in the so-called lamina cortex, and their axons terminate in the second optic neuropil, the medulla. In the lamina, axons radially extend many dendrites within each lamina cartridge that carry post-synaptic sites of tetrad synapses. The dendritic spines just beneath the lamina cortex are slightly longer than those in the deeper layers of this neuropil, and they are shortest in the proximal lamina. In the distal medulla, their terminals form irregular extensions [12] and contact many postsynaptic cells.

The oscillation in the size of two large monopolar cells, L1 and L2, have been detected in three fly species: *Musca domestica*, *Calliophora vicina* and *D*. *melanogaster* [9, 10, 13]. In the housefly, the axons of L1 and L2 monopolar cells change their girth during the day and night, and this rhythm is maintained in constant darkness (DD) and continuous light (LL). The daily pattern of plastic changes in the size of both types of interneurons is correlated with the pattern of locomotor activity of each species. Both cells are largest when locomotor activity is at its height during the day and after motor stimulation, especially in males [14].

In *D*. *melanogaster*, L1 and L2 monopolar cells swell at the beginning of the day and night and shrink in the middle of the day and night. Their axons change also shape from an inverted conical shape during the day to a cylindrical shape during the night. The fact that axons also change their girth in constant darkness indicates that an endogenous rhythm is generated by the circadian clock. The daily pattern of these changes is correlated in *D*. *melanogaster*, as in other fly species, with their locomotor activity pattern. In 12 hours of light and 12 hours of darkness (LD 12:12) conditions, the rhythm of locomotor activity of *D*. *melanogaster* exhibits two peaks, in the morning and in the evening. There were also observed changes in the size of L2 monopolar cell nuclei, which are largest at the beginning and in the middle of the day in females and males, respectively [12]. Moreover, the dendritic trees of L2 monopolar cells in *D*. *melanogaster* exhibit structural circadian plasticity [11]. Similar to axons, the L2 dendritic trees change in size and shape during the day and night. In the proximal lamina, the dendrites are longest at the beginning of the day and shorter later during the day and at night in LD 12:12. This rhythm is maintained in constant darkness (DD) but not in continuous light (LL), which indicates that this type of plasticity is controlled by the circadian clock in the brain of *D*. *melanogaster*.

Although circadian neuroplasticity in *D*. *melanogaster* L2 cells has been intensively studied, the molecular mechanism of those changes is still unknown. It has been observed that swelling and shrinking of L1 and L2 monopolar cells are not a result of osmotic shifts [9]. It is also known that the circadian plasticity of neurons requires a functional cytoskeleton and involves microtubules remodeling and actin microfilament organization. Treatment of flies with colchicine disrupted microtubules, although the shrinkage of L1 and L2 cells was not observed. In turn, the effects of cytochalasine D that disrupt actin microfilaments depend on injection time. When this chemical was injected during the night, the number of tetrad synapses and sizes of L1 and L2 monopolar cells increased [15]. In our previous study, we also found that protein synthesis is needed to increase the size of monopolar cell axons during the day, when they normally swell in the housefly, but it is not required for cell shrinking at night [14].

In the present study, we examined several proteins that might be involved in the cyclic structural plasticity of the L2 dendritic tree and the molecular mechanism of this process. One of candidate proteins is target of rapamycin (TOR), a serine-treonine kinase involved in the regulation of translation, growth and metabolism of cells. TOR and the TOR signaling pathway are highly conserved in all eukaryotes. TOR integrates different signals, including growth factors, nutrients, energy, and stress, to regulate cell growth and metabolism. Growth factors, such as insulin or insulin-like growth factor (IGFs), control TOR signaling via the PI3K pathway. Their binding to the receptor causes recruitment and phosphorylation of the insulin receptor substrate (IRS) and the recruitment of PI3K. PI3K bound to IRS converts phosphatidylinositol-4,5-phosphate (PIP2) in the cell membrane to phosphatidylinositol-3,4,5-phosphate (PIP3). In turn PIP3 recruits PDK1 and Akt to the membrane, which results in the phosphorylation and activation of Akt. The activation of Akt inhibits tuberous sclerosis complex 1 and 2 (TSC1/2), a binary-complex that negatively regulates Rheb, a small GTPase upstream of TOR kinase responsible for activation of TOR (reviewed in [16, 17]).

Because the TOR signaling pathway is responsible for regulating several cellular processes, including translation and autophagy, a process of protein degradation, we hypothesized that these two processes might be involved in the regulation of circadian plasticity of L2 monopolar cells. In addition to the L2 interneurons, we examined clock cells as another type of cells which function is known in generating circadian rhythms, including the rhythm of activity. Since locomotor activity and sleep are relatively easy to measure we used both behaviors to observe effects of the decreased TOR or autophagy protein levels in *per*-expressing clock cells on the period of the locomotor activity rhythm, the total activity, the day sleep and the night sleep in *Drosophila*.

The obtained results confirmed our hypothesis that TOR and autophagy proteins regulate the circadian plasticity of neurons and are also crucial in the clock cells affecting circadian rhythms in the behavior of *Drosophila*.

## Materials and methods

### Animals

**Fig 1:**
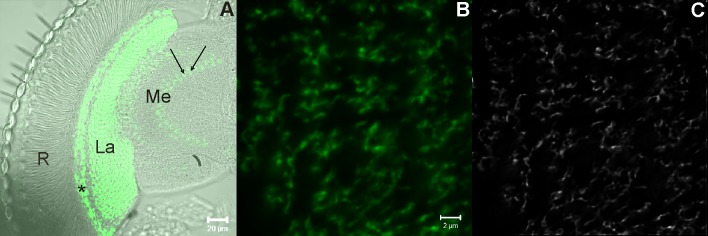
Transgenic flies (*21D-GAL4>UAS-mCD8-GFP*) with targeted GFP expression to L2 cell membranes. (A)—The L2 cell bodies are located in the lamina cortex (*), axons with dendrites in the lamina synaptic neuropil (La) and terminals of axons in the second optic neuropil (Me—medulla); R–retina, arrows show the L2 cell terminals in the medulla. (B)–Cross-section of the lamina with GFP-labeled L2 cells with dendritic trees. (C)–A deconvolved image of L2 dendritic trees for which the perimeter was measured during analyses.

Seven-day-old male Canton S wild-type flies and *per*^*01*^ mutants were used for real-time PCR experiments, and two transgenic lines, *21D-Gal4* (received from Dr. Thomas Raabe) and *UAS-mCD8-GFP* (Bloomington Drosophila Stock Centre, stock no. 5137), were used for targeted expression of GFP in L2 monopolar cell membranes (Fig 1). For analyses of the locomotor activity rhythms, *per-GAL4* (Bloomington Drosophila Stock Centre, stock no. 7127) and *UAS-Val10-GFP* (Bloomington Drosophila Stock Centre, stock no. 35786) transgenic flies were used. In addition, *UAS-Tor-RNAi* (Bloomington Drosophila Stock Centre, stock no. 34639), *UAS-Atg5-RNAi* (Bloomington Drosophila Stock Centre, stock no. 27551), *UAS-Atg7-RNAi* (Bloomington Drosophila Stock Centre, stock no. 27707), *UAS-Tsc1-RNAi* (Bloomington Drosophila Stock Centre, stock no. 31314), *UAS-Rheb-RNAi* (Bloomington Drosophila Stock Centre, stock no. 33966), *UAS-Akt1-RNAi* (Bloomington Drosophila Stock Centre, stock no. 33615) and *UAS-Pi3K-RNAi* (Bloomington Drosophila Stock Centre, stock no. 27690) transgenic lines were used for silencing *Tor*, *Atg5*, *Atg7*, *Tsc1*, *Akt1*, *Pi3K* and *Rheb* genes.

### Real-time PCR

First, we used Canton S wild-type and *per*^*01*^ flies to examine the expressions of the genes studied during the 24 h cycle. Flies were maintained on a standard cornmeal medium in a constant temperature of 25°C+/-1°C and in a light/dark regime (LD12:12) or in constant darkness (DD). They were fixed in 96% ethanol at six time points: ZT1/CT1: 1 h after lights-on/1 h after the beginning of the subjective day, ZT4/CT4: 4 h after lights-on/4 h after the beginning of the subjective day, ZT8/CT8: 8 h after lights-on/8 h after the beginning of the subjective day, ZT13/CT13: 1 h after lights-off/1 h after the beginning of the subjective night, ZT16/CT16: 4 h after lights-off/4 h after the beginning of the subjective night, ZT20/CT20: 8 h after lights-off/8 h after the beginning of the subjective night, where ZT0/CT0 represents the beginning of the day/the subjective day and ZT12/CT12 represents the beginning of the night/the subjective night. After 2-h long fixation (4°C) in 96% ethyl alcohol (POCH, Poland), brains were dissected, and RNA from 10 brains per time point was extracted using TRI® Reagent Solution (Applied Biosystems, USA) according to the manufacturer’s protocol.

Reverse transcription was performed according to the manufacturer’s protocol using a SuperScript III First-Strand Synthesis System for the RT-PCR kit (Invitrogen).

A StepOne™ Real-Time PCR System v. 2.0 (Applied Biosystems, USA) was used to quantify the relative amount of target RNA. Primers for the following genes: *Tor*, *Pi3K class 1*, *Akt1*, *Atg5* and *Atg7* and *rpl32* (Ribosomal Protein L32) were designed using Primer-BLAST software (NCBI, USA) and synthesized at GenoMed (Poland). The primer sequences utilized in the experiments are summarized in the S1 Table.

Reactions were carried out with Sybr®Green PCR Master Mix (Invitrogen, USA). The RNA of genes studied (*Tor*, *Pi3K class 1*, *Akt1*, *Atg5* and *Atg7*) was quantified as the relative fold change normalized to *rpl32* RNA. Gene expression was normalized on an arbitrary scale, where the ZT1 time point was set to 1.00 (means ± SE). The number of replicates varied in different groups and was from 3 to 7.

### Locomotor activity and sleep analysis

For the locomotor activity and sleep analyses, the following lines were used: *per-Gal4>UAS-Tor-RNAi*, *per-Gal4>UAS-Tsc1-RNAi*, *per-Gal4>UAS-Rheb-RNAi*, *per-Gal4>UAS-Atg7-RNAi*, *per-Gal4>UAS-Atg5-RNAi* and *per-GAL4>UAS-Valium10-GFP* as a control. They were used to silence the studied gene in *per* expressing cells. Instead of *per-Gal4* and various *UAS-RNAi* lines, we used the control recommended in the Flybase (http://flystocks.bio.indiana.edu/Reports/35786.html). To obtain this control line, we crossed the *per-Gal4* lines to *UAS-Valium10-GFP* and used the F1 males. These flies carried both–GAL4 and UAS constructs as well as the Valium10 –a vector used for constructing RNAi lines. The purpose of this control was to check if the position of GAL4-UAS construct in the genome has no effect on the fly’s behavior and we consider this control as genetically appropriate and valid.

The locomotor activity and sleep of the flies was recorded using a Drosophila Activity Monitoring System (Trikinetics, Waltham, MA, USA). Flies were placed in glass tubes (5 mm in diameter, 65 mm in length) with a small amount of food inside, and their locomotor activity was monitored individually. The tubes were inserted into activity monitors, which were housed inside an incubator to maintain the temperature constant (25°C), humidity and light condition. The activity of each individual male was recorded over 7 days under LD12:12 and then for 7 additional days under DD. According to the literature, sleep in *Drosophila* is defined as a period of uninterrupted behavioral immobility lasting more than 5 min [18] and in the present study walking activity of flies was recorded every 5 min. The level of activity was analyzed from the 2nd to the 7th days of recording in LD 12:12, while calculations of the total activity and the total sleep of flies in LD 12: 12 were performed using data from the second day of the experiment. In total, sleep of 178 flies was analyzed. The numbers of flies in each group was 22–32. A period of the locomotor activity rhythm was analyzed in DD using the Be Fly Excel macro, generously provided by Dr. E. Rosato. The number of flies in each group was 18–32. The locomotor activity was analyzed according to the protocol described by Rosato and Kyriacou [19]. The period of locomotor activity was recorded under free-running conditions (DD) and calculated using autocorrelation and CLEAN spectral analyses. The autocorrelation resolves the periodicity in data by comparing a time series with a time-shifted version of itself. The peak in the autocorrelation plot, that is above the 95% confidence limits, is taken as the period of the fly’s activity rhythm. The CLEAN algorithm is a computational algorithm to perform deconvolution of images created in radio astronomy. The algorithm is able to clean deeply the noise and transfer all significant features to the clean components. As a measure of significance for the different rhythmic components identified by CLEAN, Monte Carlo approach was used, by randomly shuffling the experimental data and repeating the CLEAN analysis on the new data series 100 times. The data from the simulation are plotted on a graph. The peak from the CLEAN analysis of the experimental data, that is above the 99% confidence limit, is taken as the period of the fly activity rhythm. The autocorrelation and CLEAN spectral analyses were performed using a PC software package, that has been implemented in a Python environment.

### Dendritic trees analysis

For analyses of the L2 dendritic tree perimeter, we used the progeny of crossing the *21D-Gal4* line with the *UAS-mCD8-GFP* line (Fig 1). To inhibit the expressions of the *Tor*, *Atg5* or *Atg7* genes in L2 monopolar cells, additional crosses of *21D-Gal4>UAS-mCD8-GFP* line with *UAS-Tor-RNAi*, *UAS-Atg5-RNAi* or *UAS-Atg7-RNAi* transgenic lines were performed. Seven-day-old males from the above crosses were decapitated at four time points: ZT1, ZT4, ZT13 and ZT16. The heads were fixed in 4% paraformaldehyde in 0.1 M PBS and cryoprotected overnight in 25% sucrose solution. Cryostat sections (14 μm in thickness) were prepared. To enhance GFP fluorescence in L2 cells, frozen sections were immunostained with rabbit polyclonal anti-GFP primary serum (Nouvos Biological, diluted 1:1000) followed by goat anti-rabbit secondary antibody conjugated to AlexaFluor 488 (Invitrogen, diluted 1:1000). The cryosections were mounted in Vectashield medium (Vector). Sections of the distal lamina were examined using a Zeiss Meta 510 Laser Scanning Microscope. The images were deconvolved using Huygens Professional software. Changes in the perimeter of the L2 dendritic trees were examined by tracing the outline of the dendrites and the axons of L2 cell cross-sections. Measurements were performed using ImageJ (v. 1.4 g with Java 1.6.0_05) software.

### Statistical analysis

Statistical analyses of the data were carried out using STATISTICA 12 computer software. Kruskal-Wallis nonparametric test, followed by multiple comparison test, were used to estimate the significant differences between groups in real-time PCR experiments.

Depending on a Shapiro-Wilk W test, one–way analysis of variance (ANOVA) or Kruskal-Wallis nonparametric test, followed by multiple comparison test were used to estimate the significant differences between groups in the dendritic tree analysis.

The comparison of the dendritic trees perimeter between control and experimental groups at each time point was performed using U Mann-Whitney tests or t test depending on the result of the Shapiro-Wilk W test. Similarly, significant differences in the period length of the locomotor activity rhythm, the total activity, and the sleep of the flies were estimated using U Mann-Whitney tests or t test.

## Results

### Relative expressions of *Tor*, *Pi3K class1*, *Akt1*, *Atg5* and *Atg7* genes in the brains of wild type and *per*^*01*^ flies

**Fig 2:**
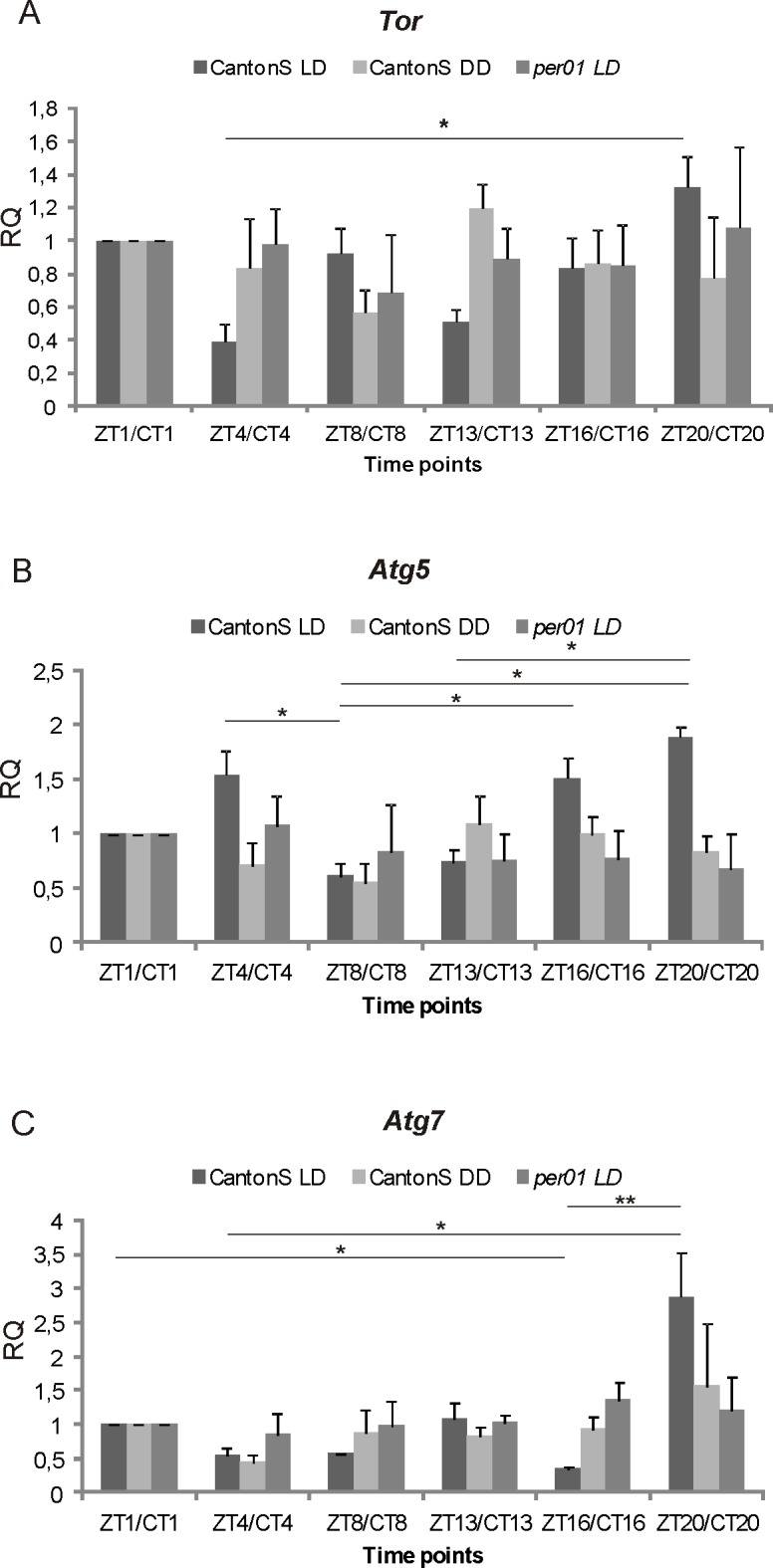
The relative level of *TOR signaling pathway and autophagy genes* RNA in the fly’s brain. **A—**The *Tor* gene RNA cycles only in the brains of Canton S in LD 12:12, reaching the highest level 4 h before the end of the night (ZT20) and the lowest 4 h after the beginning of the day (ZT4) (mean RQ +/- SE) [Kruskal-Wallis Test: H (5, N = 25) = 15,10543 p =, 0099; *post hoc* multiple comparison test: * < 0.05]. **B—**The relative level of *Atg5* RNA in the brain of Canton S male flies, held in LD 12:12 or in DD and in *per*^*01*^ mutants in LD 12:12 (mean RQ +/- SE). The *Atg5* RNA cycles in the brains of Canton S in LD 12:12, reaching the highest level 4 h after the beginning of the day (ZT4) and 4 h before the end of the night (ZT20) [Kruskal-Wallis test: H (5, N = 35) = 23,34957 p =, 0003, *post hoc* multiple comparison test: * < 0.05]. **C**—The relative level of *Atg7* RNA in the brain of Canton S male flies, held in LD 12:12 or in DD and in *per*^*01*^ mutants in LD 12:12 (mean RQ +/- SE). The *Atg7* RNA cycles in the brain of Canton S in LD 12:12. The highest level was detected 4 h before the end of the night (ZT20) and at the beginning of the day (ZT1) [Kruskal-Wallis test: H (5, N = 31) = 23,07912 p =, 0003, *post hoc* multiple comparison test: * < 0.05; ** < 0.01].

The relative expression of the *Tor* gene in the brains of wild-type flies kept in LD12:12, was the highest 4 h before the end of the night (ZT20). The RNA level of this gene dropped during the day (by 24.58% at ZT1), reached the lowest value at ZT4 (by 70.81%) and increased later during the night. This rhythm was not detected when the flies were kept in DD or in the *per*^*01*^ mutants kept in LD 12:12 (Fig 2A).

The relative expressions of *Atg5* gene in the brains of Canton S flies raised in LD 12:12 showed a bimodal pattern of the rhythm (Fig 2B). The *Atg5* RNA reached the highest level in the middle of the day (ZT4–53.13% higher than in ZT1) and 4 h before the end of the night (ZT20–89.10% higher than ZT1) and dropped at the beginning of the day (ZT1) and later during the day (ZT8) and at the beginning of the night (ZT13) (Fig 2B). The highest level of *Atg7* RNA was observed in 4 h before the end of the night (ZT20) and at the beginning of the day (ZT1). At ZT4, the level of *Atg7* RNA was 43.92% lower than at ZT1, and at ZT16, it was 64.77% lower than at ZT1 (Fig 2C). In DD there were no statistically significant differences in RNA levels of these genes. In the brains of *per*^*01*^ mutants, the levels of RNA of both genes was the same at all time points studied.

*Akt1* and *Pi3K class 1* RNAs did not show any changes in the brains of *D*. *melanogaster* maintained neither in LD12:12 nor in DD. The levels of their RNA were also constant in the *per*^*01*^ mutants (S1 Fig).

### Locomotor activity of flies with silenced expression of TOR signaling pathway and autophagy genes in *per-*expressing cells

**Fig 3:**
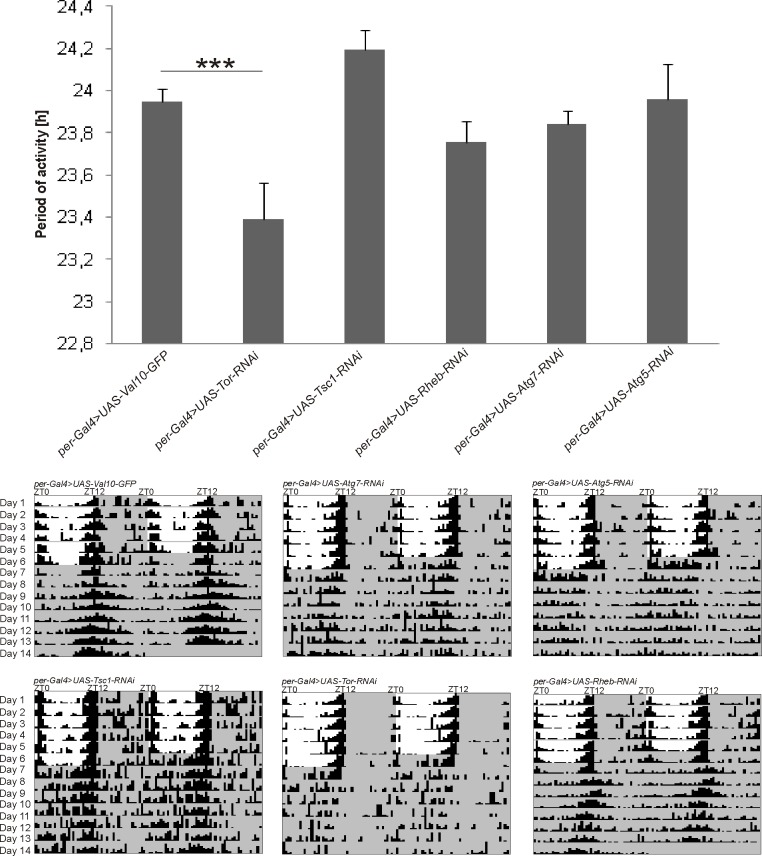
Periods of the locomotor activity rhythm of flies after silencing the expressions of studied genes. *Tor*, *Tsc1*, *Rheb*, *Atg5* and *Atg7* genes expression was silenced in *per*-positive cells. Period of locomotor activity rhythm was measured in DD conditions (mean +/- SE). Representative actograms were presented. As a control, the progeny of *per*-Gal4 and UAS-Val10-GFP crossing was used. After silencing the *Tor* gene, the period of locomotor activity rhythm was significantly shorter than in the control [U Mann-Whitney Test, *** for p≤0,001]. Silencing the *Tsc1* gene caused a slight lengthening in the period of the rhythm.

**Fig 4:**
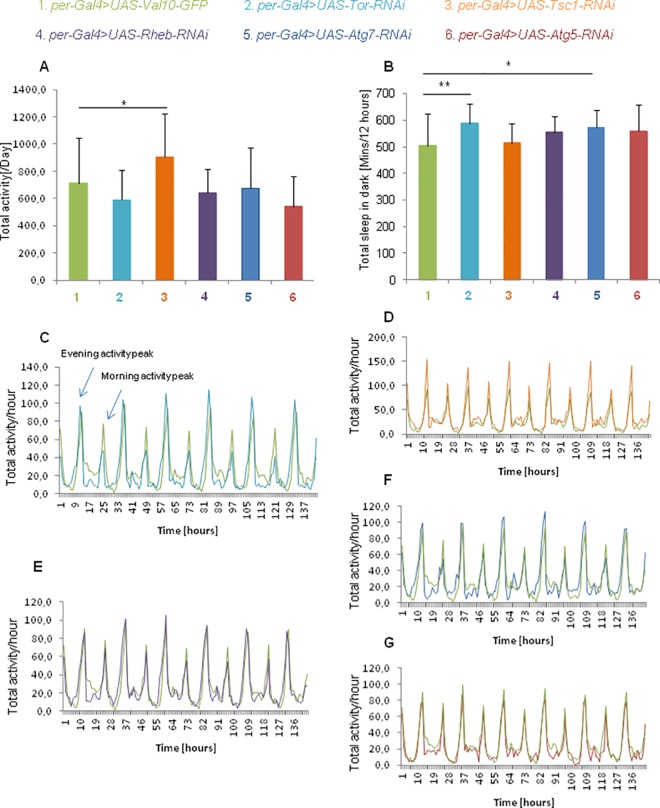
Sleep and activity of flies with silenced expression of TOR signaling pathway and autophagy genes. (A)—The total activity of flies recorded on the second day of experiment (mean +/- SD). Silencing the *Tsc1* gene in *per*-positive cells increased the total activity of the flies [U Mann-Whitney Test, * for p≤0,05]. (B)—Total sleep of flies in the dark phase (mean +/- SD). The flies with silenced expressions of *Tor* or *Atg7* gene had lengthened sleep durations in the dark [U Mann-Whitney Test, ** for p≤0,01, * for p≤0,5]. (C)–The sleep metrics of flies with silenced expression of the *Tor* gene in *per*-positive cells (x axis shows the time in hours). These flies were less active than control flies during the morning peak of activity but were more active during the evening peaks. (D)—The sleep metrics of flies with silenced expression of the *Tsc1* gene in *per*-positive cells. The experimental flies were more active than control flies during the entire recorded period, regardless of the time of day. (E)—The sleep metrics of flies with silenced expression of the *Rheb* gene in *per*-positive cells. Experimental flies were less active during morning peaks of activity in comparison to control flies. (F)—The sleep metrics of flies with silenced expression of the *Atg7* gene in *per*-positive cells. (G)—The sleep metrics of flies with silenced expression of the *Atg5* gene in *per*-positive cells.

After silencing the *Tor* gene in *per*-expressing cells, the period of the locomotor activity rhythm was significantly decreased by 0.5 h compared with the control insects. In turn, after the silencing of *Tsc1* gene, the period was slightly longer. The silencing of the *Atg5* and *Atg7* genes in *per-*positive cells had no influence on the period of locomotor activity rhythm (Fig 3). The decreases in TOR and ATG7 or ATG5 also affected both the robustness and pattern of the circadian rhythm of locomotor activity in DD as shown on actograms of flies with silenced *Tor*, *Atg5* or *Atg7* genes and of the control flies (Fig 3). The rhythm was weaker than in the control, and the peak at the beginning of the subjective night present in controls was not observed in experimental flies (Fig 3). Moreover, the decreased levels of TOR and TSC1 proteins changed the activity of flies. In case of TSC1, the total activity of the flies increased after silencing of *Tsc1*, while the decrease in TOR diminished the morning peak but enhanced the evening peak of activity (Fig 4). There were no statistically significant changes in total sleep during the light phase in LD 12:12 (S2 Fig), but silencing of the *Tor* or *Atg7* genes caused a lengthening of the sleep duration during the dark phase of LD 12:12 (Fig 4B).

### Morphometric analysis of the L2 dendritic tree perimeter

**Fig 5:**
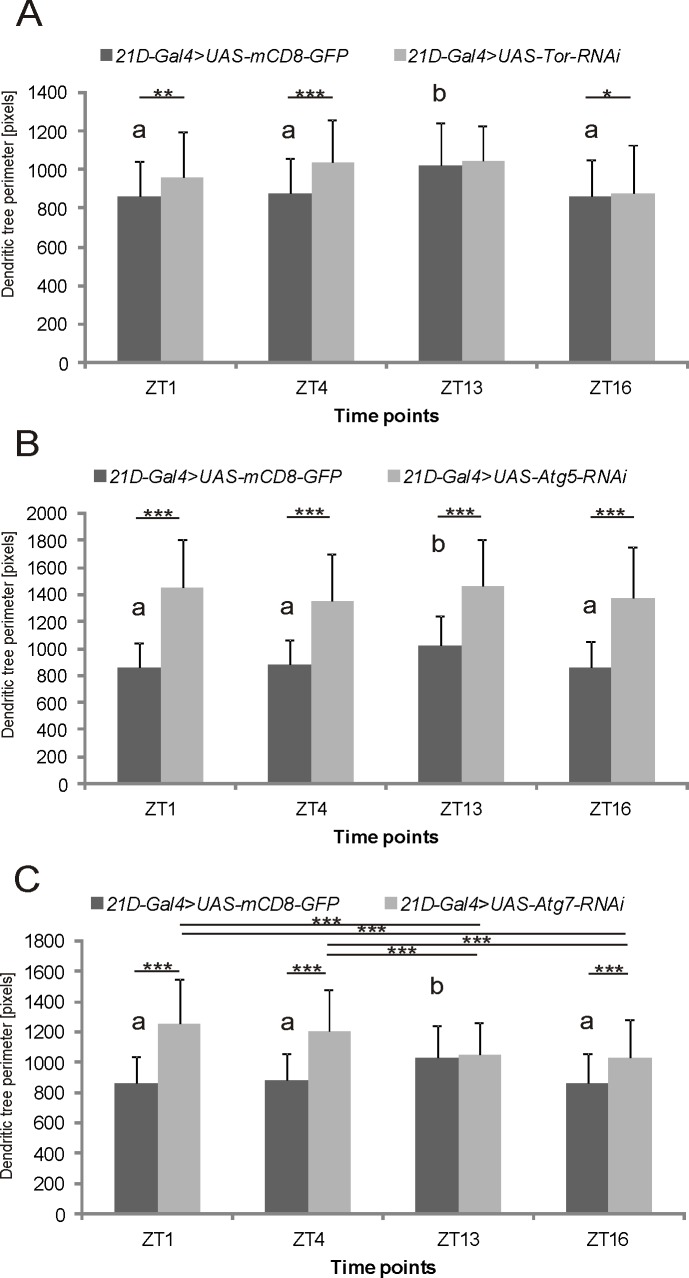
The perimeter of L2 cell dendritic trees after silencing *Tor*, *Atg5* and *Atg7* genes. After silencing the *Tor* (A) or *Atg5* (B) under control of the 21D-promoter, the daily rhythm of the L2 cell dendritic tree size was abolished (mean +/- SD) [*Tor*: ANOVA, p>0.05; *Atg5*: Kruskal-Wallis Test: H (3, N = 401) = 7,722824 p =, 0521], while in case of *Atg7* (C), the pattern of the daily rhythm of the L2 dendritic trees was changed. The dendritic trees were largest at the beginning of the day, and their size decreased during the day and at night [Kruskal-Wallis Test: H (3, N = 412) = 40.63845 p = 0.0000; multiple comparison test, ***—p≤0.01]. The differences between time points in control flies are indicated with “a” and “b” letters. The dendritic trees are largest at the beginning of the night, in ZT13—a [Kruskal-Wallis test: H (3, N = 423) = 23,31382 p =, 0000; multiple comparison test, the difference between ZT13 and ZT4—p≤0.01, differences among ZT13 and ZT1 and ZT13 and ZT16 p≤0.001].

The L2 cell dendritic trees, which were measured in the distal part of the lamina, showed a daily rhythm in their size and shape changes, with a peak at the beginning of the night (ZT13). Silencing of the *Tor* gene in L2 cells under control of the 21D promoter abolished the rhythm of dendritic tree plasticity (Fig 5A). Similar results were observed after silencing *Atg5* in L2 monopolar cells (Fig 5B). In contrast, silencing of *Atg7* changed the pattern of the rhythm in the L2 cell dendritic tree perimeter. The largest dendritic tree outline was observed at the beginning of the day (ZT1), was decreased during the day (ZT4) by 3,61% compared to at ZT1, and further decreased at night by 15,87% (ZT13) and 17.96% (ZT16) compared to ZT1 (Fig 5C). The decreases in *Tor*, *Atg5* and *Atg7* mRNA levels enlarged the L2 dendritic trees at all time point studied, except at ZT13 for *Tor* and *Atg7*. The silencing of *Akt1* and *Pi3K* in L2 cells was lethal for larvae and pupae, respectively.

## Discussion

The obtained results indicate that both the circadian clock and light affect the expression of *Tor* and autophagy genes. Moreover, the TOR signaling pathway and autophagy are involved in the regulation of the locomotor activity rhythm, the activity level, sleep and circadian plasticity of L2 monopolar cells. *Tor*, *Atg5* and *Atg7* genes showed cyclic expressions in the brain of Canton S flies kept in LD 12:12, and these rhythms were abolished in the brain of *per*^*01*^ mutants. The rhythms were not maintained in DD, and this indicates that their expression is probably masked in DD because *per* is needed for their rhythmic expression in LD. The microarray results obtained by Claridge-Chang et al [20] detected 158 genes with the circadian expression in the head of *D*. *melanogaster* in LD conditions, however, *Tor*, *Atg5* and *Atg7* were not among them. Similarly, McDonald and Rosbash [21] and Ceriani et al [22] did not find circadian expression of *Tor*, *Atg5* and *Atg7* in the fly’s head in DD. Our results indicate that *Tor*, *Atg5* and *Atg7* have the daily rhythm of expression in the brain of *D*. *melanogaster*. The differences in results obtained by other authors and in the present study may results from using whole heads for analyses but not dissected brains, knowing that heads contain fat body and include retinas, so their genes affect the gene expression profile of the brain. It is not surprising, taking into consideration the results obtained from isolated PDF-expressing clock neurons by Kula-Eversole et al [23]. They have found that *Atg5* and *Atg7* genes are differently expressed in large and small PDF-expressing ventral lateral neurons (LNv) of flies raised in LD conditions. *Atg5* was downregulated at ZT12 vs ZT0 in large LNv, but its expression was about two times higher in ZT12 vs ZT0 in small LNv. *Atg7* gene change fold was 2.56 in ZT12 in the small LNv, but in the large LNv, mRNA of this gene was downregulated at ZT18 vs ZT6 and vs ZT0. We found that the majority of cells in the brain show different patterns of *Atg5* and *Atg7* mRNA cycling in LD 12:12 than in small and large LNv.

The daily pattern of *Tor* expression in the fly brains has not yet been described. The circadian activity of this enzyme has been detected in the mammalian brain in the suprachiasmatic nuclei (SCN), the mammalian circadian clock site. Cao et al. [24] showed that, in mouse SCN, the activity of the mTOR kinase is cyclic in DD because its activity marker, the phosphorylated S6 ribosomal protein (pS6), is high during the subjective day while the expression of S6 is constant. In addition, light simulation upregulates mTOR activation, and the application of rapamycin, an inhibitor of mTOR, to the lateral ventricle of the brain blocks the effect of light. In turn, light pulses applied during the subjective day do not affect the activity of p70 S6K kinase [25]. The results obtained in the present study suggest a similar mechanism in the brain of *D*. *melanogaster*. *Tor* expression is not cyclical in DD, but the decrease of TOR affects circadian rhythms of locomotor activity and neuronal plasticity. *Tor* seems to be a clock-controlled gene, but light regulates its expression.

On the other hand, it seems that TOR is not only controlled by the circadian clock and light but that it also regulates circadian rhythms by itself. Our results showed that decreasing the expression of *Tor* in *per*-expressing cells causes significant shortening of a period of the *Drosophila* locomotor activity rhythm. The results obtained by other authors confirm our findings. The overexpression of S6K, the main target of TOR complexes, TORC1, causes the lengthening of a period of the locomotor activity rhythm [26]. Cao et al. [27] also observed an influence of mTOR kinase activity on the mouse photic entrainment and phase of the locomotor activity rhythm. In turn, administration of rapamycin in the early subjective night (CT15) significantly attenuated the phase-delay effect of light, but in the absence of light, the early night infusion of rapamycin did not significantly affect the clock phase. In turn, the disruption of mTOR signaling at CT22 significantly increased the light-induced phase advance.

Protein synthesis is needed to increase axon sizes of L1 and L2 monopolar cells during the day when they normally swell in the day/night cycle in the housefly, *Musca domestica* [14]. The swelling of axons is likely correlated with the enlargement of dendritic trees, and this process depends on TOR because silencing of *Tor*, a key regulator of protein translation, in the lamina L2 monopolar cells in *Drosophila* disrupts the rhythm of the L2 dendritic tree. This indicates that TOR inhibits the extension of dendrites during the day and at the end of the night.

TOR protein is also involved in processes such as local protein translation in dendrites (reviewed in [28]) and synaptic plasticity [29]. Jaworski et al. [30] reported that the mTOR signaling pathway promotes growth and arborization of cultured hippocampal neurons. One of the pathways that activates mTOR and is involved in neuronal growth, survival and neuroplasticity is the PI3K/AKT pathway. The activation of this pathway increases dendrite branching, while inhibition of mTOR by rapamycin or RNAi diminishes the number of dendrites and the complexity of dendritic trees of the cultured hippocampal neurons [30]. Our results proved that TOR signaling pathway is also responsible for regulating circadian structural plasticity, as observed in the lamina L2 interneurons of *D*. *melanogaster*.

Moreover, we found that autophagy is also involved in the regulation of the circadian structural plasticity of L2 interneurons. Autophagy is an evolutionary conservative process, in which parts of cytoplasm containing proteins and damaged organelles are surrounded by a double membrane forming autophagosomes and fusing with lysosomes for degradation of its content [31]. Macroautophagy is a constitutively active and highly efficient process in healthy neurons [32], and its cyclic activity has been observed in liver, heart and skeletal muscles of mice (reviewed in [33]). We found that RNA levels of autophagy genes *Atg5* and *Atg7* oscillate during the day, and both rhythms are bimodal in LD 12:12 in the brain of *D*. *melanogaster*. However, their RNA levels cycle in a different manner. *Atg5* RNA was the most abundant in the middle of the day and in the middle of the night, while the RNA of *Atg7* was most abundant in the middle of the night (ZT20) and at the beginning of the day and dropped significantly early during the night. The high level of *Atg5* RNA at ZT4, ZT16 and ZT20 might be involved in the shrinkage of L1 and L2 monopolar cells in *D*. *melanogaster* during napping and sleeping [10], respectively, because of the withdrawal of proteins and membranes responsible for dendrite extension and the enlargement of the girth of axons. In contrast, ATG7 has the same effect as TOR on the sizes of L2 dendritic trees, decreasing their sizes during the day and at the end of the night, because the silencing of *Tor*, *Atg5* and *Atg7* enlarged the L2 dendritic tree.

The daily changes in autophagy have also been found in the retina of mice with two peaks, in the middle of the day and in the middle of the night, when the highest ratio of LC3-II to LC3-I and the most autophagy related proteins and Atg5-Atg12 complexes were observed [34].

Autophagy in the nervous system plays an important role in maintaining homeostasis in neurons. Disturbances in this process can lead to neurodegeneration and neuronal cell death [35]. It has been observed that mice lacking Atg7, specifically in the central nervous system, show behavioral defects, including abnormal limb-clasping reflexes and a reduction in coordinated movement, and they die within 28 weeks of birth. Atg7 deficiency causes massive neuronal losses in the cerebral and cerebellar cortices [36].

In our model, silencing of *Atg7* in L2 cells caused changes in the pattern of daily plasticity of L2 cell dendritic trees. The size of the L2 dendritic tree was the largest at the beginning of the day, and not at the beginning of the night, as in the control. In turn, silencing of the *Atg5* gene abolished this rhythm, indicating the role of autophagy in the regulation of circadian neuronal plasticity.

The roles of autophagy in starvation, proteins and organelles degradation, and programmed cell death are well known, but this process also seems to be involved in the remodeling of cells during development as well as during the day in the adult brain. Shen and Ganetzky [37] showed that autophagy promotes the development of neuromuscular junctions in *D*. *melanogaster* and *atg1*, *atg2*, *atg6*, and *atg18* mutants exhibit reduced growth of neuromuscular junctions and a reduced number of synaptic buttons. It has also been suggested that autophagy is activated during the early neurite growth of cultured cortical neurons and negatively regulates its growth. The inhibition of autophagy by *atg7* small interfering RNA (siRNA) caused elongation of axons, while activation of autophagy by rapamycin suppressed their growth [38].

Although autophagy was thought to be a non-selective process of protein degradation, there are some data that suggest that autophagy may act selectively in some developmental events, such as for the degradation of cell-surface GABA receptors in *Caenorhabditis elegans* [39] or for the selective elimination of several maternally derived germ P granule components in somatic cells during *C*. *elegans* embryogenesis [40].

Our results showed the role of autophagy in the circadian neuronal plasticity in the visual system of *D*. *melanogaster*. In the present study, we examined the involvement of TOR and two autophagy proteins only in LD 12:12; however, because neuronal plasticity of L2 interneurons is also maintained in constant darkness [11], TOR, ATG5 and ATG7 are important for circadian plasticity of neurons. Protein synthesis and degradation as well as membrane turnover must be involved in the lengthening and shortening of dendrites. In L2 interneurons TOR and ATG5 seems to be crucial to maintain the rhythm of shrinking and extending the L2 dendritic tree while ATG7 is involved in the pattern of the rhythm. Its high level at the end of night and at the beginning of the day shortens dendrites but low level lengthens them. TOR controls translation and autophagy as one of the processes responsible for protein degradation, and it is regulated by the TOR kinase. TOR may regulate PER level in L2 cells, however, PER does not cycle in those cells [41] but affects their morphology. In *per*^01^ mutants the L2 dendrites are shorter than in wild-type flies [11]. This indicates that TOR is crucial for many processes in neurons, including plasticity, as shown in case of the L2 interneurons but also in clock cells that generates rhythms in neuronal processes and in behavior. The role of TOR in regulating synaptic plasticity, which has been described as long-term potentiation (LTP) and long-term depression (LTD), has already been reported [42, 43]. It seems that the TOR signaling pathway may be crucial in the regulation of all aspects of plasticity in the brain.

In the *per*-expressing clock cells, TOR could be a cellular sensor of external stimuli, for example light, and may regulate activity/sleep ratio, promoting activity. The silencing of *Tor Atg5* or *Atg7* in *Drosophila* decreased activity and extended sleep during the night. In this case TOR may activate *Atg7* in the middle of night while *Atg5* expression might be inhibited in the middle of the day (nap) and activated in the middle of the night (sleep). In addition TOR but not ATG5 and ATG7, affects the molecular mechanism of the clock since the decreased level of TOR shortens the period of the locomotor activity rhythm. Our studies showed that the TOR signaling pathway and ATG5 and ATG7 are crucial for the regulation of cellular processes and can play various functions in different cell types in the brain.

## Supporting information

S1 Fig The relative level of *Akt1* and *Pi3K class 1* genes RNA in the fly’s brain. A—The relative level of *Akt1* RNA in the brain of Canton S male flies, held in LD 12:12 or in DD and in *per*^*01*^ mutants in LD 12:12 (mean RQ +/- SE). The *Akt1* RNA level is constant in Canton S and *per*^*01*^ flies in LD 12:12 and in DD. B—The relative level of the *Pi3K class 1* gene RNA in the brain of Canton S male flies, held in LD 12:12 or in DD and in *per*^*01*^ mutants in LD 12:12 (mean RQ +/- SE). The *PI3K class 1* RNA level does not cycle in the brains of insect studied.
(TIF)

S2 Fig Sleep in the light phase of flies with silenced expression of TOR signaling pathway and autophagy genes (mean +/- SD). There were no statistically significant differences in the length of sleep in the light phase between the experimental and control flies.
(TIF)

S1 File Results obtained from measuring the L2 cell dendritic tree perimeter. (XLSX)

S2 File Results of period length of the locomotor activity rhythm of flies with silenced expression of TOR signaling pathway and autophagy genes in *per*-positive cells. (XLSX)

S3 File Real time PCR results after analyzing gene expression in the brain of Canton S flies in DD. (XLS)

S4 File Real time PCR results after analyzing gene expression in the brain of Canton S flies in LD12:12. (XLSX)

S5 File Real time PCR results after analyzing gene expression in the brain of *per*^*01*^ flies in LD12:12. (XLSX)

S6 File Results of measuring total activity of flies with silenced expression of TOR signaling pathway and autophagy genes in *per*-positive cells. (XLSX)

S7 File Results of measuring total sleep in dark and light phases of flies with silenced expression of TOR signaling pathway and autophagy genes in *per*-positive cells. (XLSX)

S1 Table The primers used in the experiments. (DOCX)

## References

[1] Stanewsky R. Genetic analysis of the circadian system in *Drosophila melanogaster* and mammals. J. Neurobiol. 2003 1;54(1):111–47. 10.1002/neu.10164 12486701

[2] Tataroglu O, Emery P. The molecular ticks of the *Drosophila* circadian clock. Curr Opin Insect Sci. 2015 2 1;7:51–57. 10.1016/j.cois.2015.01.002 26120561 PMC4480617

[3] Pyza E, Górska-Andrzejak J. External and internal inputs affecting plasticity of dendrites and axons of the fly's neurons. Acta Neurobiol Exp (Wars). 2008;68(2):322–33. 18511964 10.55782/ane-2008-1698

[4] Górska-Andrzejak J, Damulewicz M, Pyza E. Circadian changes in neuronal networks. Curr Opin Insect Sci. 2015 2;7:76–81. 10.1016/j.cois.2015.01.005 32846686

[5] Górska-Andrzejak J, Salvaterra PM, Meinertzhagen IA, Krzeptowski W, Görlich A, Pyza E. Cyclical expression of Na+/K+-ATPase in the visual system of *Drosophila melanogaster*. J. Insect. Physiol. 2009 5;55(5): 459–68. 10.1016/j.jinsphys.2009.02.003 19428365 PMC2721802

[6] Damulewicz M, Rosato E, Pyza E. Circadian regulation of the Na+/K+-ATPase alpha subunit in the visual system is mediated by the pacemaker and by retina photoreceptors in *Drosophila melanogaster*. PLoS One. 2013; 8(9): e73690 10.1371/journal.pone.0073690 24040028 PMC3769360

[7] Górska-Andrzejak J, Makuch R, Stefan J, Görlich A, Semik D, Pyza E. Circadian expression of the presynaptic active zone protein Bruchpilot in the lamina of *Drosophila melanogaster*. Dev. Neurobiol. 2013 1;73(1):14–26. 10.1002/dneu.22032 22589214

[8] Damulewicz M, Pyza E. The clock input to the first optic neuropil of *Drosophila melanogaster* expressing neuronal circadian plasticity. PLoS One. 2011; 6(6): e21258 10.1371/journal.pone.0021258 21760878 PMC3124489

[9] Pyza E, Meinertzhagen IA. Monopolar cells axons in the first optic neuropil of the housefly, *Musca domestica L*., undergo daily fluctuations in diameter that have a circadian basis. J. Neurosci. 1995 1;15(1):407–18. 7823145 10.1523/JNEUROSCI.15-01-00407.1995 PMC6578271

[10] Pyza E, Meinertzhagen IA. Daily rhythmic changes of cell size and shape in the first neuropil in *Drosophila melanogaster*. J. Neurobiol. 1999 7;40(1):77–88. 10398073 10.1002/(sici)1097-4695(199907)40:1<77::aid-neu7>3.0.co;2-0

[11] Weber P, Kula–Eversole E, Pyza E. Circadian control of dendrite morphology in the visual system of *Drosophila melanogaster*. PLoS One. 2009; 4(1), e4290 10.1371/journal.pone.0004290 19173003 PMC2628732

[12] Górska–Andrzejak J, Kellar A, Raabe T, Kilianek Ł, Pyza E. Structural daily rhythms in GFP-labeled neurons in the visual system of *Drosophila melanogaster*. Photochem. Photobiol. Sci. 2005 9;4(9):721–6. 10.1039/b417023g 16121283

[13] Pyza E, Cymborowski B. Circadian rhythms in behaviour and in the visual system of the bowlfly *Calliphora vicina*. J. Insect Phisiol. 2001 7 15;47(8):897–904.

[14] Kula E, Pyza E. Effects of locomotor stimulation and protein synthesis inhibition on circadian rhythms in size changes of L1 and L2 interneurons in the fly’s visual system. Develop. Neurobiol. 2007 9 15;67(11):1433–42. 10.1002/dneu.20518 17497696

[15] Pyza E. Dynamic structural changes of synaptic contacts in the visual system of insect. Microsc Res Tech. 2002 8 15;58(4):335–44. 10.1002/jemt.10141 12214300

[16] Parisi F, Riccardo S, Daniel M, Saqcena M, Kundu N, Pession A, et al *Drosophila* insulin and target of rapamycin (TOR) pathways regulate GSK3 beta activity to control Myc stability and determine Myc expression in vivo. BMC Biol. 2011 9 27;9:65 10.1186/1741-7007-9-65 21951762 PMC3235970

[17] Wullschleger S, Loewith R, Hall MN. TOR signaling in growth and metabolism. Cell. 2006 2 10;124(3):471–84. 10.1016/j.cell.2006.01.016 16469695

[18] Huber R, Hill SL, Holladay C, Biesiadecki M, Tononi G, Cirelli C. Sleep homeostasis in *Drosophila melanogaster*. Sleep. 2004 6 15;27(4):628–39. 15282997 10.1093/sleep/27.4.628

[19] Rosato E, Kyriacou CP. Analysis of locomotor activity rhythms in Drosophila. Nat Protoc. 2006;1(2):559–68. 10.1038/nprot.2006.79 17406282

[20] Claridge-Chang A, Wijnen H, Naef F, Boothroyd C, Rajewsky N, Young MW. Circadian regulation of gene expression systems in the Drosophila head. Neuron. 2001 11 20;32(4):657–71. 11719206 10.1016/s0896-6273(01)00515-3

[21] McDonald MJ, Rosbash M. Microarray analysis and organization of circadian gene expression in Drosophila. Cell. 2001 11 30;107(5):567–78. 11733057 10.1016/s0092-8674(01)00545-1

[22] Ceriani MF^1^, Hogenesch JB, Yanovsky M, Panda S, Straume M, Kay SA. Genome-wide expression analysis in Drosophila reveals genes controlling circadian behavior. J Neurosci. 2002 11 1;22(21):9305–19. 12417656 10.1523/JNEUROSCI.22-21-09305.2002 PMC6758054

[23] Kula-Eversole E, Nagoshi E, Shang Y, Rodriguez J, Allada R, Rosbash M. Surprising gene expression patterns within and between PDF-containing circadian neurons in *Drosophila*. Proc Natl Acad Sci U S A. 2010 7 27;107(30):13497–502. 10.1073/pnas.1002081107 20624977 PMC2922133

[24] Cao R, Anderson FE, Jung YJ, Dziema H, Obrietan K. Circadian regulation of mammalian target of rapamycin signaling in the mouse suprachiasmatic nucleus. Neuroscience. 2011 5 5;181:79–88. 10.1016/j.neuroscience.2011.03.005 21382453 PMC3102430

[25] Cao R, Lee B, Cho HY, Saklayen S, Obrietan K. Photic regulation of the mTOR signaling pathway in the suprachiasmatic circadian clock. Mol. Cell Neurosci. 2008 7;38(3):312–24. 10.1016/j.mcn.2008.03.005 18468454 PMC2590753

[26] Zheng X, Seghal A. AKT and TOR signaling set the pace of the circadian pacemaker. Curr. Biol. 2010 7 13;20(13):1203–8. 10.1016/j.cub.2010.05.027 20619819 PMC3165196

[27] Cao R, Li A, Cho HY, Lee B, Obrietan K. Mammalian target of rapamycin signaling modulates photic entrainment of the suprachiasmatic circadian clock. J. Neurosci. 2010 5 5;30(18):6302–14 10.1523/JNEUROSCI.5482-09.2010 20445056 PMC2896874

[28] Wang DO, Martin KC, Zukin RS. Spatially restricting gene expression by local translation at synapses. Trends Neurosci. 2010 4;33(4):173–82. 10.1016/j.tins.2010.01.005 20303187 PMC3503250

[29] Cammalleri M, Lütjens R, Berton F, King AR, Simpson C, Francesconi W, et al Time-restricted role for dendritic activation of the mTOR-p70S6K pathway in the induction of late-phase long-term potentiation in the CA1. Proc. Natl. Acad. Sci. USA 2003 11 25;100(24):14368–73. 10.1073/pnas.2336098100 14623952 PMC283598

[30] Jaworski J, Spangler S, Seeburg DP, Hoogenraad CC, Sheng M. Control of dendritic arborization by the phosphoinositide-3'-kinase-Akt-mammalian target of rapamycin pathway. J. Neurosci. 2005 12 7;25(49):11300–12. 10.1523/JNEUROSCI.2270-05.2005 16339025 PMC6725892

[31] Yang Z, Klionsky DJ. Eaten alive: a history of macroautophagy. Nat. Cell Biol. 2010 9;12(9): 814–822. 10.1038/ncb0910-814 20811353 PMC3616322

[32] Boland B, Kumar A, Lee S, Platt FM, Wegiel J, Yu WH. Autophagy induction and autophagosome clearance in neurons: relationship to autophagic pathology in Alzheimer's disease. J. Neurosci. 2008 7 2;28(27):6926–37. 10.1523/JNEUROSCI.0800-08.2008 18596167 PMC2676733

[33] Ma D, Li S1, Molusky MM, Lin JD. Circadian autophagy rhythm: a link between clock and metabolism? Trends Endocrinol. Metab. 2012 7;23(7):319–25. 10.1016/j.tem.2012.03.004 22520961 PMC3389582

[34] Yao J, Jia L, Shelby SJ, Ganios AM, Feathers K, Thompson DA, et al Circadian and noncircadian modulation of autophagy in photoreceptors and retinal pigment epithelium. Invest. Ophthalmol. Vis Sci. 2014 5;55(5):3237–46. 10.1167/iovs.13-13336 24781939 PMC4037936

[35] Lee JA. Neuronal autophagy: a housekeeper or a fighter in neuronal cell survival? Exp Neurobiol. 2012 3;21(1):1–8. 10.5607/en.2012.21.1.1 22438673 PMC3294068

[36] Komatsu M, Waguri S, Chiba T, Murata S, Iwata J, Tanida I, et al Loss of autophagy in the central nervous system causes neurodegeneration in mice. Nature 2006 6 15;441(7095):880–4 10.1038/nature04723 16625205

[37] Shen W, Ganetzky B. Autophagy promotes synapse development in *Drosophila*. J. Cell Biol. 2009 10 5;187(1):71–9. 10.1083/jcb.200907109 19786572 PMC2762098

[38] Ban BK, Jun MH, Ryu HH, Jang DJ, Ahmad ST, Lee JA. Autophagy negatively regulates early axon growth in cortical neurons. Mol. Cell Biol. 2013 10;33(19):3907–19. 10.1128/MCB.00627-13 23918799 PMC3811863

[39] Rowland AM, Richmond JE, Olsen JG, Hall DH, Bamber BA. Presynaptic terminals independently regulate synaptic clustering and autophagy of GABA_A_ receptors in *Caenorhabditis elegans*. J. Neurosci. 2006 2 8;26(6):1711–20. 10.1523/JNEUROSCI.2279-05.2006 16467519 PMC6793639

[40] Zhang Y, Yan L, Zhou Z, Yang P, Tian E, Zhang K, et al SEPA-1 mediates the specific recognition and degradation of P granule components by autophagy in *C*. *elegans*. Cell 2009 1 23;136(2):308–21. 10.1016/j.cell.2008.12.022 19167332

[41] Damulewicz M, Loboda A, Bukowska-Starkova, Jozkowicz A, Dulak J, Pyza E. Clock and clock-controlled genes are differently expressed in the retina, lamina and in selected cells of the visual system of *Drosophila melanogaster*. Front. Cell. Neurosci. 2015 9 15; 9:353 10.3389/fncel.2015.00353 26441524 PMC4569741

[42] Sui L, Wang J, Li BM. Role of the phosphoinositide 3-kinase-Akt-mammalian target of the rapamycin signaling pathway in long-term potentiation and trace fear conditioning memory in rat medial prefrontal cortex. Learn Mem. 2008 10 2;15(10):762–76. 10.1101/lm.1067808 18832563

[43] Hou L, Klann E. Activation of the phosphoinositide 3-kinase-Akt-mammalian target of rapamycin signaling pathway is required for metabotropic glutamate receptor-dependent long-term depression. J. Neurosci. 2004 7 14;24(28):6352–61. 10.1523/JNEUROSCI.0995-04.2004 15254091 PMC6729543

