# Supplementary figures and images for: TOR signaling pathway and autophagy are involved in the regulation of circadian rhythms in behavior and plasticity of L2 interneurons in the brain of Drosophila melanogaster
Source: PLoS One. 2017 Feb 14;12(2):e0171848. doi: 10.1371/journal.pone.0171848 (PMC5308838; doi:10.1371/journal.pone.0171848)

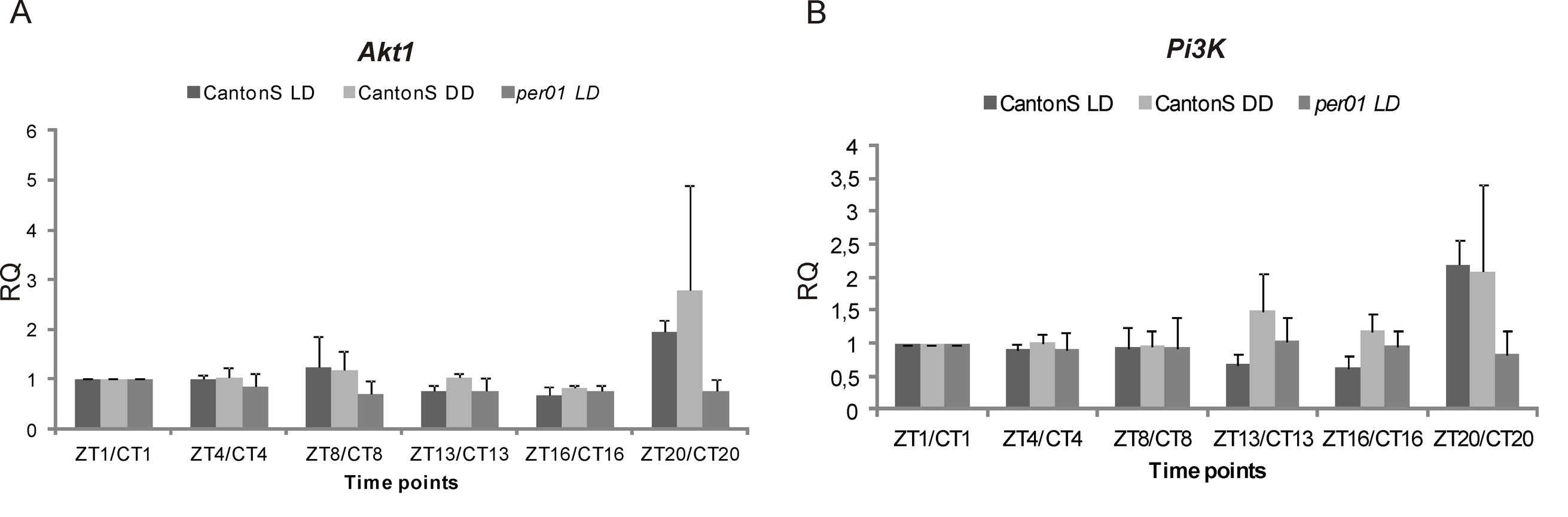

Supplement: S1 Fig — A—The relative level of Akt1 RNA in the brain of Canton S male flies, held in LD 12:12 or in DD and in per01 mutants in LD 12:12 (mean RQ +/- SE). The Akt1 RNA level is constant in Canton S and per01 flies in LD 12:12 and in DD. B—The relative level of the Pi3K class 1 gene RNA in the brain of Canton S male flies, held in LD 12:12 or in DD and in per01 mutants in LD 12:12 (mean RQ +/- SE). The PI3K class 1 RNA level does not cycle in the brains of insect studied. (TIF) [file pone.0171848.s001.tif]

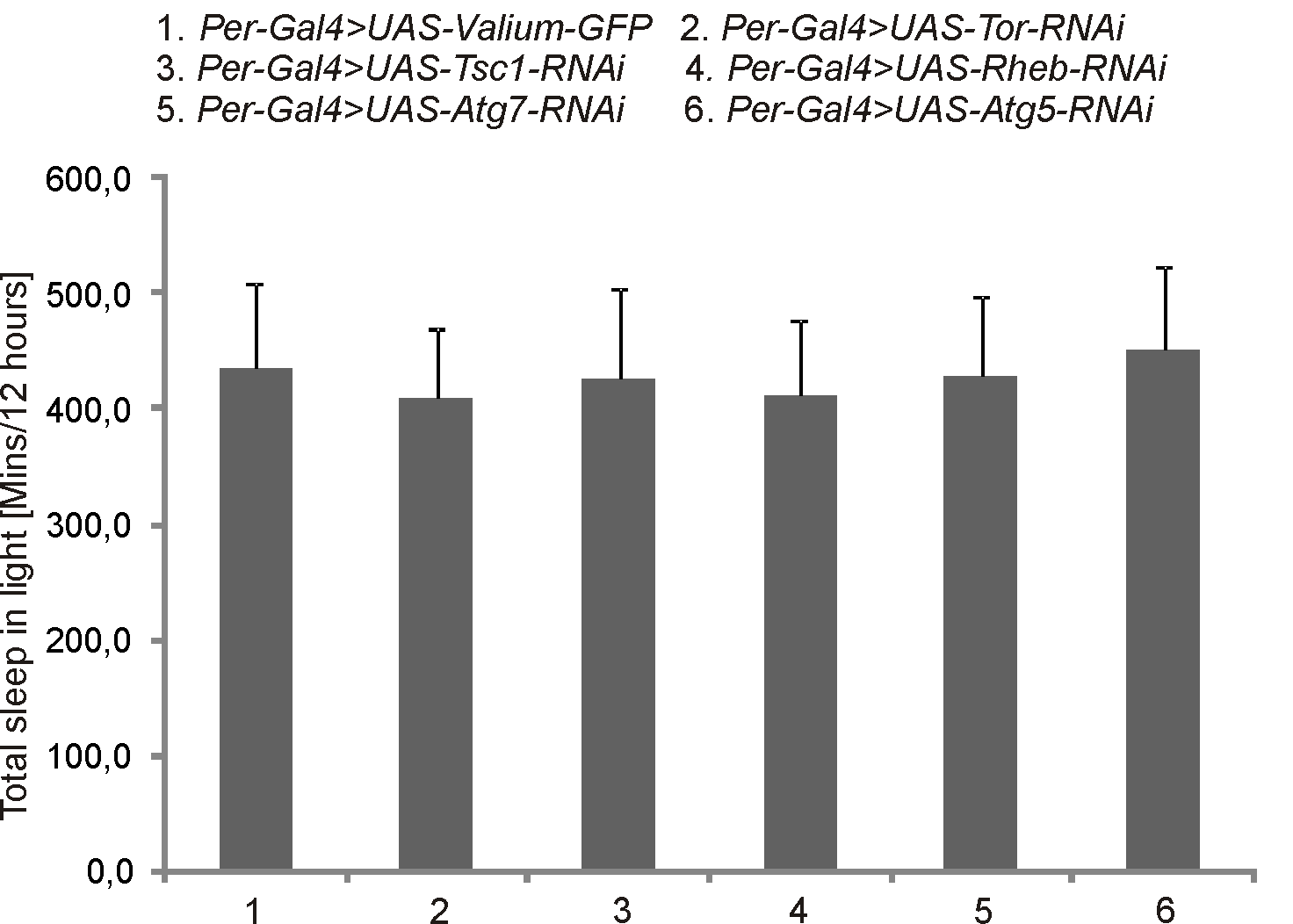

Supplement: S2 Fig — There were no statistically significant differences in the length of sleep in the light phase between the experimental and control flies. (TIF) [file pone.0171848.s002.tif]
