# Supplementary material for: TOR signaling pathway and autophagy are involved in the regulation of circadian rhythms in behavior and plasticity of L2 interneurons in the brain of Drosophila melanogaster
Source: PLoS One. 2017 Feb 14;12(2):e0171848. doi: 10.1371/journal.pone.0171848 (PMC5308838; doi:10.1371/journal.pone.0171848)
Supplement: S1 Table — (DOCX) [file pone.0171848.s010.docx]

| Gene name | Primer sequence | Product length (base pairs) |
| --- | --- | --- |
| *Tor* forward | 5′-GAGTGAGTTCCTGGCTCCCCGG-3′ | 131 |
| *Tor* reverse | 5′-GGGCGAAATGCCCTTCCTGGC-3′ |  |
| *Atg5* forward | 5’-TCGTACATATCCTGGCAGGTTCGC-3’ | 106 |
| *Atg5* reverse | 5’-GAAGTCGCAGGGGCGTTCCAT-3’ |  |
| *Atg7* forward | 5'-CATTCCGCTATAGGCACCAT-3' | 172 |
| *Atg7* reverse | 5'-CGGCAAAGGAGAGAACAAAG-3' |  |
| *Akt1* forward | 5’-CCGCAGTGCAGTAGGGAAAGCAAA-3’ | 133 |
| *Akt1* reverse | 5’-GCGACCTAGCAGCGCAACATGT-3’ |  |
| *Pi3K* forward | 5’-GCTATAAAGGCGAGGGCTGGCC-3’ | 182 |
| *Pi3K* reverse | 5’-ACGCGGCACCGATGTCTCATC-3’ |  |
| *rpl32* forward | 5′-AGAAGCGCAAGGAGATTGTC-3′ | 233 |
| *rpl32* reverse | 5′-ATGGTGCTGCTATCCCAATC-3 |  |

Supplementary table 1. The primers used in the experiments.
